# Emergence of *Mycobacterium orygis*–Associated Tuberculosis in Wild Ruminants, India

**DOI:** 10.3201/eid2903.221228

**Published:** 2023-03

**Authors:** Megha Sharma, Karikalan Mathesh, Premanshu Dandapat, Asok Kumar Mariappan, Ravi Kumar, Soni Kumari, Vivek Kapur, Sushila Maan, Naresh Jindal, Nitish Bansal, Riyaz Kadiwar, Abhishek Kumar, Nitin Gupta, A.M. Pawde, A.K. Sharma

**Affiliations:** Indian Council of Agricultural Research—Indian Veterinary Research Institute, Izatnagar, India (M. Sharma, K. Mathesh, A.K. Mariappan, R. Kumar, S. Kumari, A.M. Pawde, A.K. Sharma);; Indian Council of Agricultural Research—Indian Veterinary Research Institute, Eastern Regional Station, Kolkata, India (P. Dandapat);; Pennsylvania State University, University Park, Pennsylvania, USA (V. Kapur);; Lala Lajpat Rai University of Veterinary and Animal Sciences, Hisar, India (S. Maan, N. Jindal, N. Bansal);; Sakkarbaug Zoological Garden, Junagarh, India (R. Kadiwar, A. Kumar);; Bandhavgarh National Park, Madhya Pradesh, India (N. Gupta)

**Keywords:** tuberculosis and other mycobacteria, Indian bison, *Mycobacterium orygis*, spotted deer, India

## Abstract

Tuberculosis caused by *Mycobacterium orygis* was detected in 2 spotted deer from a wildlife sanctuary in western India and an Indian bison from a national park in central India. Nationwide surveillance is urgently required to clarify the epidemiology of the *Mycobacterium tuberculosis* complex at the human–livestock–wildlife interface.

Tuberculosis (TB) caused by *Mycobacterium orygis* has been reported in humans, cattle, and, rarely, wild animals in India (*1*–*3*). We report 3 cases of *M. orygis*–associated TB in wild animals from among 85 unexplained deaths screened as part of disease investigations during February 2016–March 2020, which also revealed cases of suppurative bronchopneumonia (n = 32), TB caused by *M. tuberculosis* or *M. bovis* (n = 29), verminous pneumonia (n = 9), fungal granulomas (n = 6), and neoplasms (n = 6).

In February 2016, two adult free-range spotted deer (a male [case 1] and a female [case 2]) were found dead in Girnar Wildlife Sanctuary, Gujarat, western India. Postmortem examination revealed nonuniform, multifocal, coalescing pale-yellow nodules embedded in the parenchyma of the lungs with caseated yellowish-white material and enlarged liver and mesenteric lymph nodes with surface nodules. In January 2017, an emaciated adult male bison (case 3) was found dead at Bandhavgarh National Park, Madhya Pradesh, central India. Similar to the deer, the bison had variable-sized white caseous nodules on the visceral pleura, superficial lung parenchyma, and pulmonary lymph nodes.

To investigate the causative agent, tissues from the lungs, liver, and lymph nodes were collected on ice and 10% neutral buffered formalin and processed for histopathology, Ziehl-Neelsen staining, and culture isolation. Histopathologic examination of these tissues revealed large granulomas with extensive caseous necrosis and multiple calcified areas surrounded by epithelioid cells, lymphocytes, giant cells, and fibroblasts (most abundant in case 3). Acid-fast bacilli were abundant (50–75/oil immersion field), both extracellularly and within the macrophages. We cultured all samples in triplicate in Löwenstein–Jensen media with glycerol and in Löwenstein–Jensen with sodium pyruvate, which revealed moist, smooth, and granular colonies (*4*). Primary screening of bacterial isolates by single-tube multiplex PCR that targets the 16S rRNA, specific for the *Mycobacterium* genus, and MPB70 genes, specific for members of MTBC, confirmed that the isolates were MTBC (*5*). We performed further PCR on the MTBC-positive samples to determine the presence or absence of genomic regions of difference (RD4 and RD9) using published primers (*6*); this testing indicated the absence of RD9 and presence of RD4, thus excluding the possibility of *M. tuberculosis*, *M. canetti*, *M. bovis*, or *M. bovis* BCG in all 3 cases.

To determine the exact species of MTBC involved and their genetic similarities with strains affecting livestock and humans circulating in India, we performed paired-end whole-genome sequencing on the Illumina MiSeq platform (https://www.illumina.com). The presence of standard genetic markers for *M. orygis* (RD1, RD4, and Rv044c) and the absence of RD9 and RD12 confirmed our sequences as *M. orygis*. We submitted the whole-genome data generated to the National Center for Biotechnology Information Sequence Read Archive database under accession nos. SRX15482219 (case 1), SRX6969199 (case 2), and SRX6969201 (case 3). 

We phylogenetically compared the sequences generated in this study with other available *M. orygis* sequences. The phylogenetic branching patterns suggest that the isolate from the case 1 spotted deer was genetically closer to the isolate recovered from the bison (case 3) than the case 2 deer isolate (Figure). The average pairwise difference across all the isolates in this study was 272; it was 73 between the isolates. The restricted diversity observed among several of the newly described isolates, including those recovered from among free-living wildlife, is noteworthy and requires future investigations.

**Figure:**
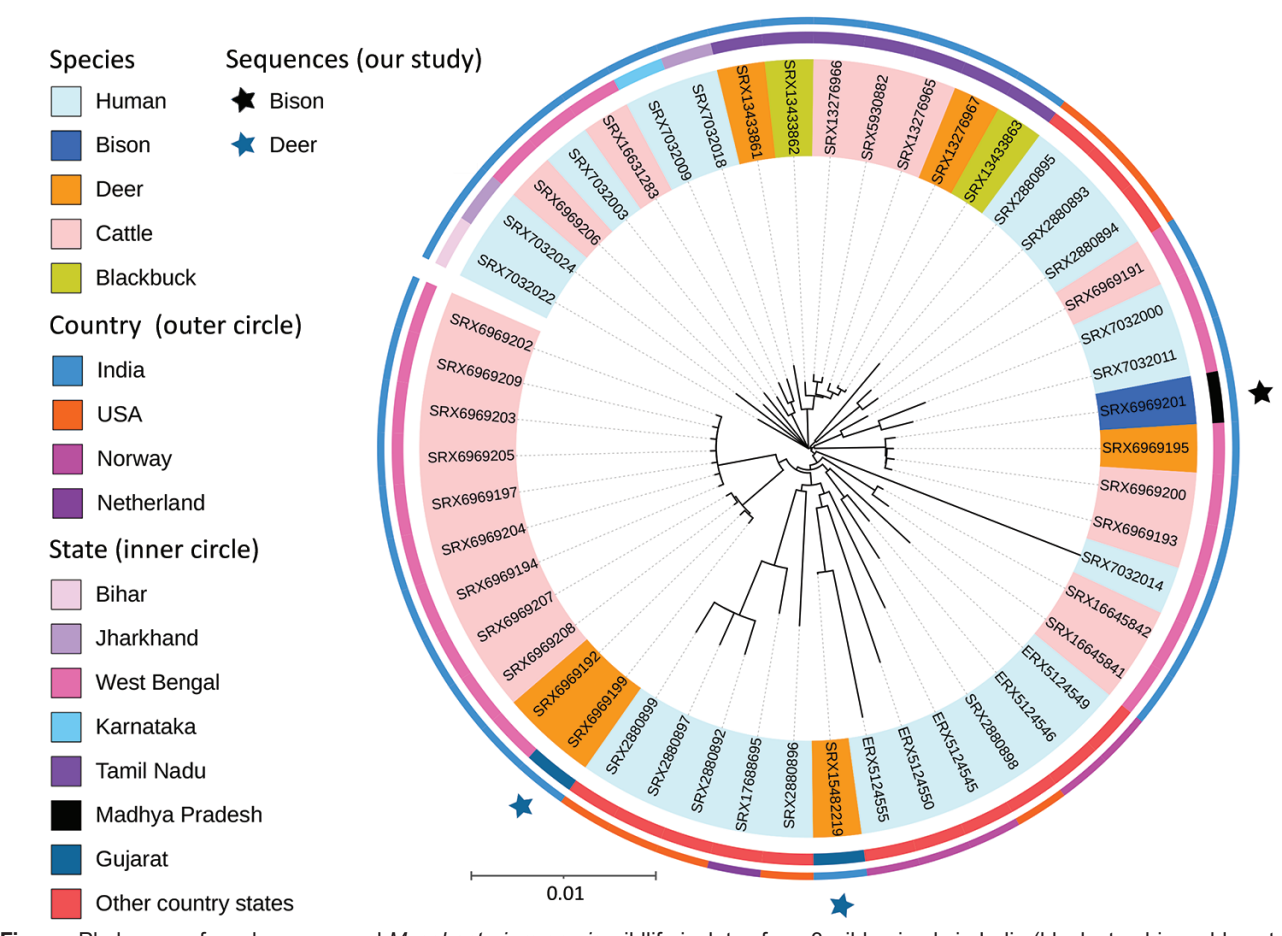
Phylogeny of newly sequenced Mycobacterium orygis wildlife isolates from 3 wild animals in India (black star, bison; blue stars, deer) and reference sequences. The outer circle shows the distribution of isolates in India, Norway, the Netherlands, and the United States. The inner circle shows the statewise distribution within countries. Shading of branch labels corresponds to different species. Scale bar of 0.01 indicates 1 change for every 100 nucleotides.

Recently, *M. orygis* has emerged as a zoonotic threat in south Asia (*7*); multispecies cases have been reported in India involving humans, dairy cattle, and wild ungulates (*1*–*3*,*7*,*8*). Studies from Nepal and Bangladesh have also revealed the circulation of *M. orygis* in free-ranging wild animals and cattle, which indicates the possibility of *M. orygis* in the India multihost wildlife system (*9*). Reports on the transmission of *M. orygis* infection from an India-origin farm worker to cattle in New Zealand (*10*) and the confirmation of *M. orygis* in 10 human patients in south Asia (*2*) imply endemicity in the region, highlighting the urgent need for genomic epidemiologic investigations.

We report the circulation of *M. orygis* in free-ranging wildlife populations in India, suggesting an unexplored threat to wildlife conservation in regions where various endangered species coexist. In this study, the transmission dynamics of *M. orygis* are unknown; however, spillover and spillback episodes might have occurred because of the shared space and resources at the livestock-wildlife-human interface. In India, human population explosion has led to encroachment on forest areas and shrinking wildlife habitats, which has increased the threat of pathogen transmission among wildlife, livestock, and humans. Although the epidemiology has not been defined, phylogenetic analysis in our study and previous reports indicate that *M. orygis* appears to be circulating in wild animal, human, and livestock populations in India. In light of the World Health Organization End TB Strategy, nationwide screening and continuous surveillance under the umbrella of the One Health approach should be conducted to combat this deadly zoonotic disease.

Appendix Additional information about the emergence of *Mycobacterium orygis*–associated tuberculosis in wild ruminants, India
