# Supplementary material for: Emergence of Mycobacterium orygis–Associated Tuberculosis in Wild Ruminants, India
Source: Emerg Infect Dis. 2023 Mar;29(3):661–3. doi: 10.3201/eid2903.221228 (PMC9973683; doi:10.3201/eid2903.221228)
Supplement: Appendix — Additional information about the emergence of Mycobacterium orygis–associated tuberculosis in wild ruminants, India [file 22-1228-Techapp-s1.pdf]

Article DOI: https://doi.org/10.3201/eid2903.221228

Emergence of *Mycobacterium orygis*–Associated Tuberculosis in Wild Ruminants, India

Appendix

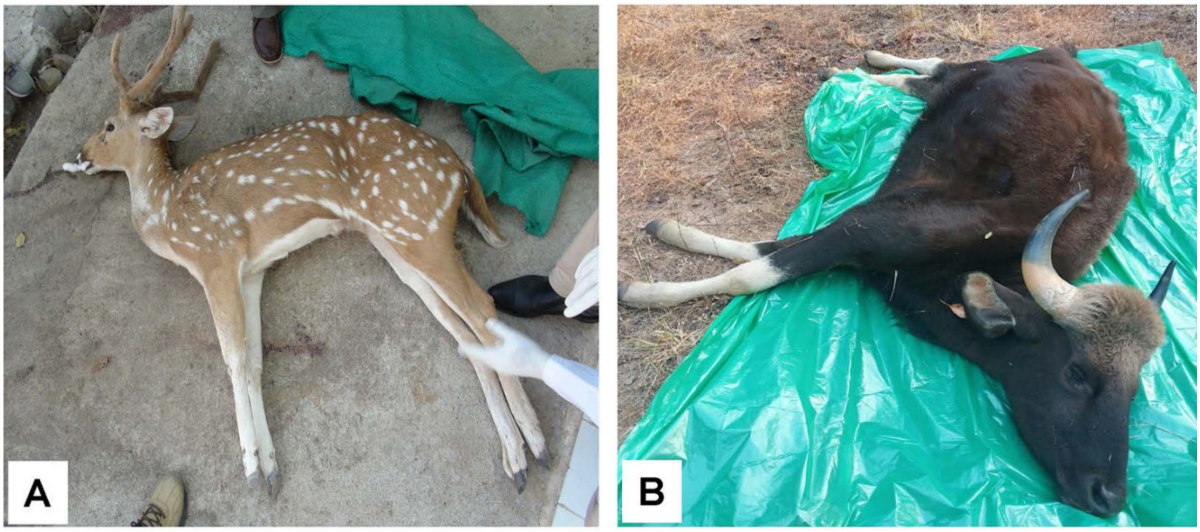

Appendix Figure 1. Weak and debilitated carcasses of spotted deer (A) and bison (B) affected with TB

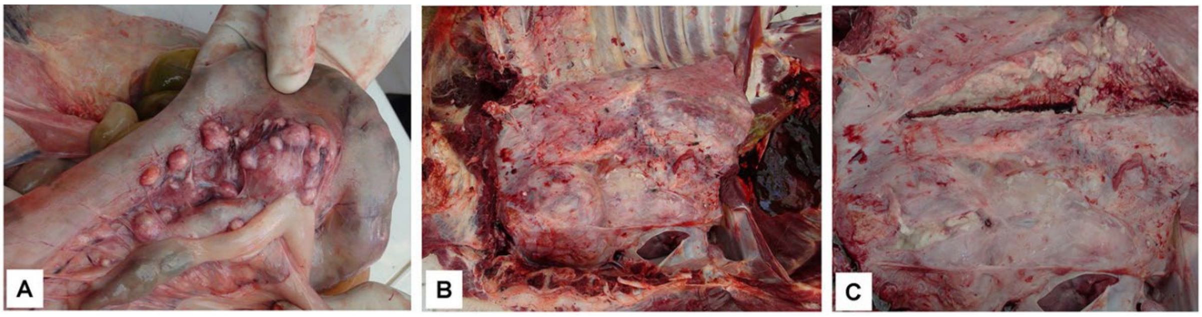

Appendix Figure 2. Spotted deer, enlarged mesenteric lymph nodes (A) showing tuberculous nodules. Multiple poorly demarcated coalescing nodules filled with pale-white caseous material (B & C)

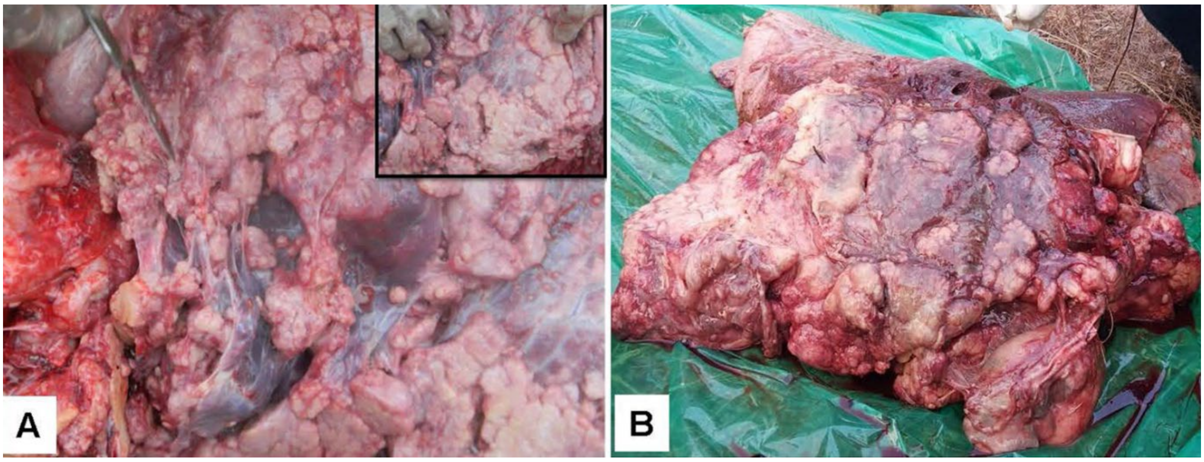

Appendix Figure 3. Bison, multifocal to coalescing, poorly demarcated, variable-sized white caseous nodules on the visceral pleura (A) and the lung parenchyma (B)

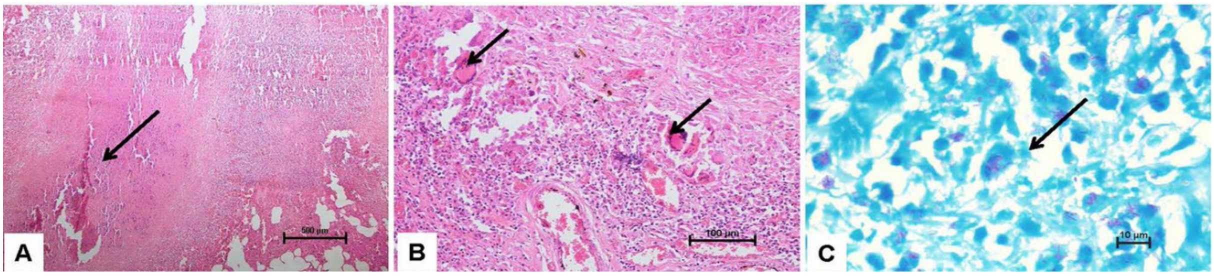

Appendix Figure 4. Spotted deer, lung showing extensive caseo-calcified areas (arrow) surrounded by inflammatory cells with associated emphysema (A) H&E ×40. Higher magnification showing epithelioid cells and giant cells in the periphery of granuloma (B) (arrows) H&E ×200 and acid-fast bacilli inside macrophages (C) ZN ×1000

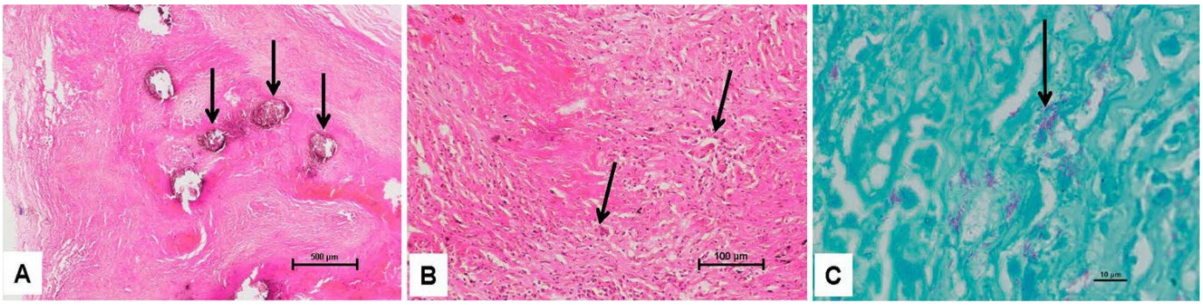

Appendix Figure 5. Bison, lung showing multiple caseo-calcified granulomas (arrows) in the visceral pleura with associated fibrosis (A) H&E ×40. Higher magnification showing attempts of giant cells formation (arrows) in the periphery of granuloma with extensive fibroblasts proliferation (B) H&E ×200 and acid-fast bacteria (arrow) in the caseative granuloma (C) ZN × 1000

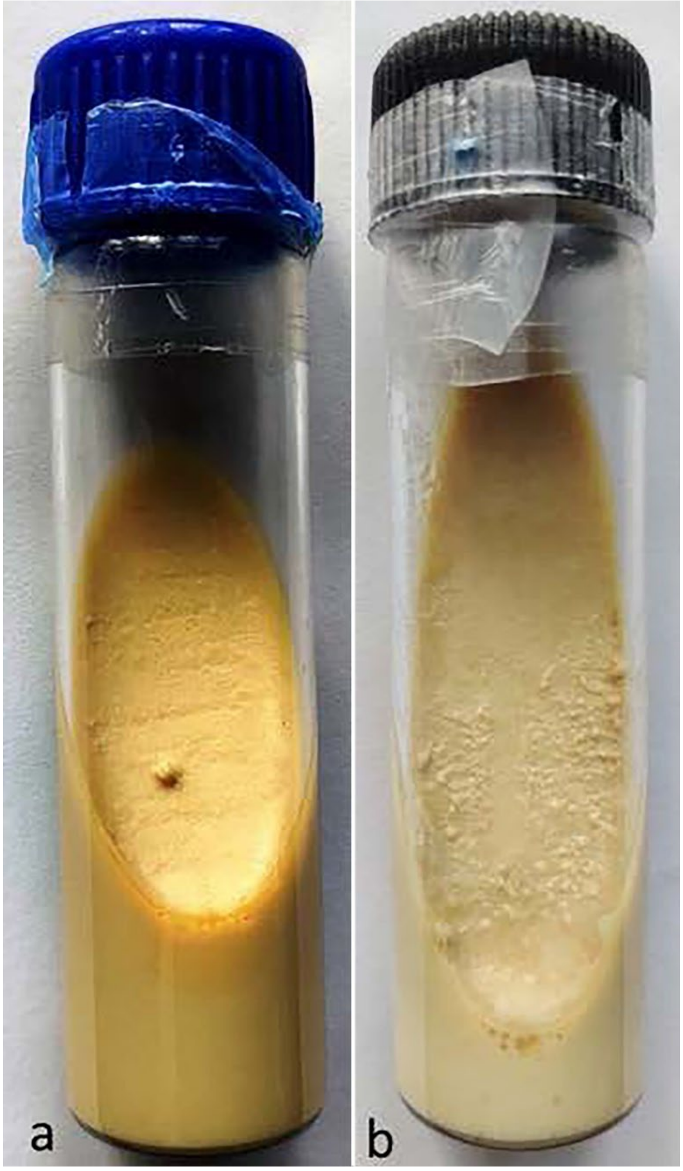

Appendix Figure 6. Granular and moist colonies of *M. orygis* from spotted deer (a) and bison (b) in Löwenstein–Jensen medium (LJ) supplemented with pyruvate for 4 weeks
